# Selecting intervention content to target barriers and enablers of recognition and response to deteriorating patients: an online nominal group study

**DOI:** 10.1186/s12913-022-08128-6

**Published:** 2022-06-10

**Authors:** Duncan Smith, Martin Cartwright, Judith Dyson, Jillian Hartin, Leanne M. Aitken

**Affiliations:** 1 School of Health Sciences, City, University of London, Northampton Square, London, EC1V 0HB UK; 2 Patient Emergency Response & Resuscitation Team (PERRT), University College London Hospitals NHS Foundation Trust, Euston Road, London, NW1 2BU UK; 3 Reader in Implementation Science, Birmingham City University, Westbourne Road, Edgbaston, Birmingham, B15 3TN UK; 4 School of Nursing and Midwifery, Griffith University, Nathan, QLD 4111 Australia

**Keywords:** Clinical deterioration, Critical care, Vital signs, Behavioural research, Group processes, Consensus, Nursing

## Abstract

**Background:**

Patients who deteriorate in hospital wards without appropriate recognition and/or response are at risk of increased morbidity and mortality. Track-and-trigger tools have been implemented internationally prompting healthcare practitioners (typically nursing staff) to recognise physiological changes (e.g. changes in blood pressure, heart rate) consistent with patient deterioration, and then to contact a practitioner with expertise in management of acute/critical illness. Despite some evidence these tools improve patient outcomes, their translation into clinical practice is inconsistent internationally. To drive greater guideline adherence in the use of the National Early Warning Score tool (a track-and-trigger tool used widely in the United Kingdom and parts of Europe), a theoretically informed implementation intervention was developed (targeting nursing staff) using the Theoretical Domains Framework (TDF) version 2 and a taxonomy of Behaviour Change Techniques (BCTs).

**Methods:**

A three-stage process was followed: 1. TDF domains representing important barriers and enablers to target behaviours derived from earlier published empirical work were mapped to appropriate BCTs; 2. BCTs were shortlisted using consensus approaches within the research team; 3. shortlisted BCTs were presented to relevant stakeholders in two online group discussions where nominal group techniques were applied. Nominal group participants were healthcare leaders, senior clinicians, and ward-based nursing staff. Stakeholders individually generated concrete strategies for operationalising shortlisted BCTs (‘applications’) and privately ranked them according to acceptability and feasibility. Ranking data were used to drive decision-making about intervention content.

**Results:**

Fifty BCTs (mapped in stage 1) were shortlisted to 14 (stage 2) and presented to stakeholders in nominal groups (stage 3) alongside example applications. Informed by ranking data from nominal groups, the intervention was populated with 12 BCTs that will be delivered face-to-face, to individuals and groups of nursing staff, through 18 applications.

**Conclusions:**

A description of a theory-based behaviour change intervention is reported, populated with BCTs and applications generated and/or prioritised by stakeholders using replicable consensus methods. The feasibility of the proposed intervention should be tested in a clinical setting and the content of the intervention elaborated further to permit replication and evaluation.

**Supplementary Information:**

The online version contains supplementary material available at 10.1186/s12913-022-08128-6.

## Contributions to the literature

- To improve the recognition and/or response to deteriorating patients (by nursing staff), a range of intervention components may be required, including training and different Behaviour Change Techniques delivered using a range of concrete strategies.
- Behaviour Change Techniques, used to optimise the physical and social environment, could be delivered in acute hospital wards at the point of care.
- It may be more suitable to deliver some appropriate BCTs in a workshop setting, particularly when the end-users are healthcare staff and delivery of the techniques involves prompting reflection on the consequences of enacting or not enacting specific (clinical) behaviours, and/or making plans for future behaviour.
- Strategies for delivering BCTs within the ward setting were broadly favoured by clinical stakeholders (i.e. considered more acceptable and/or feasible) over alternate strategies for delivery in workshops. The acceptability of different approaches requires further examination during feasibility testing.

## Background

**Fig. 1:**
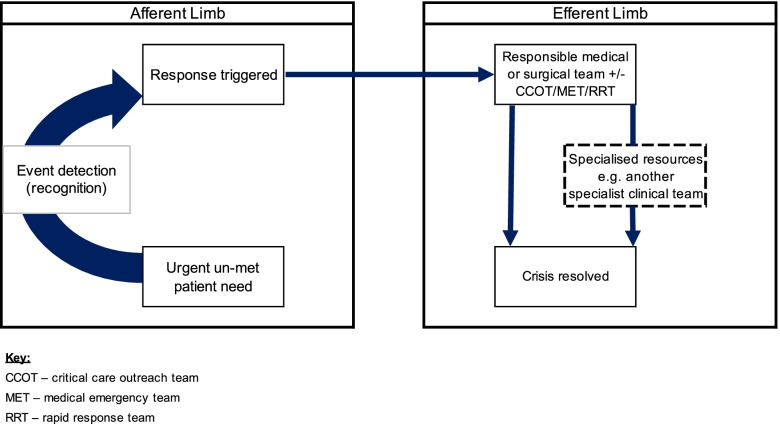
Conceptual model of the Rapid Response System (RRS). Adapted from: DeVita et al. [4]

Clinical deterioration has been defined as a change in the condition of a patient from one clinical state to a worse clinical state with an increased risk of morbidity or mortality [1]. Hospitalised patients who deteriorate in a ward setting, without recognition or an appropriate response, are at risk of Serious Adverse Events (SAEs) such as unplanned admission to the Intensive Care Unit (ICU), cardiac arrest, and/or death [2, 3]. To facilitate recognition of, and response to, patient deterioration, Rapid Response Systems (RRSs) have been implemented within acute hospitals internationally [4]. At the system level, RRSs typically include an ‘afferent limb’ (the recognition arm) and an ‘efferent limb’ (the response arm) (Fig. 1). However, there is often variation between organisations in how RRSs are operationalised [4, 5].

Changes in vital signs (e.g. heart rate, respiratory rate, blood pressure) are present in more than 50% of patients who suffer SAEs [6–8]. To strengthen the afferent limb of the RRS, track-and-trigger tools have been implemented internationally. These tools (which may be paper-based or embedded within an electronic health record), permit healthcare practitioners (frequently nursing staff) to record vital signs, providing a signal when the vital signs breach pre-determined criteria (i.e. when the vital signs fall outside of acceptable ranges). When criteria are breached, staff are prompted to escalate care; that is, to increase the frequency of vital signs monitoring and to contact a more senior colleague or a practitioner with expertise in the management of critical illness (e.g. a doctor or a nurse from critical care outreach team or equivalent) [9, 10]. In the UK and parts of Europe, the National Early Warning Score (NEWS) has been widely implemented and its predictive performance validated [11–13]. The NEWS comprises six routinely recorded vital signs [14]. For each vital sign, a score is applied (range 0–3) depending on the level of physiological derangement. The scores are then combined, and for patients requiring supplemental oxygen a further two points added, to produce the total NEWS (range 0–20). The higher the NEWS, the greater the risk to the patient of SAE and the more senior the practitioner to whom care should be escalated [14]. The use of early warning scores (like NEWS) and accompanying escalation of care protocols are associated with improved patient outcomes [15].

**Fig. 2:**
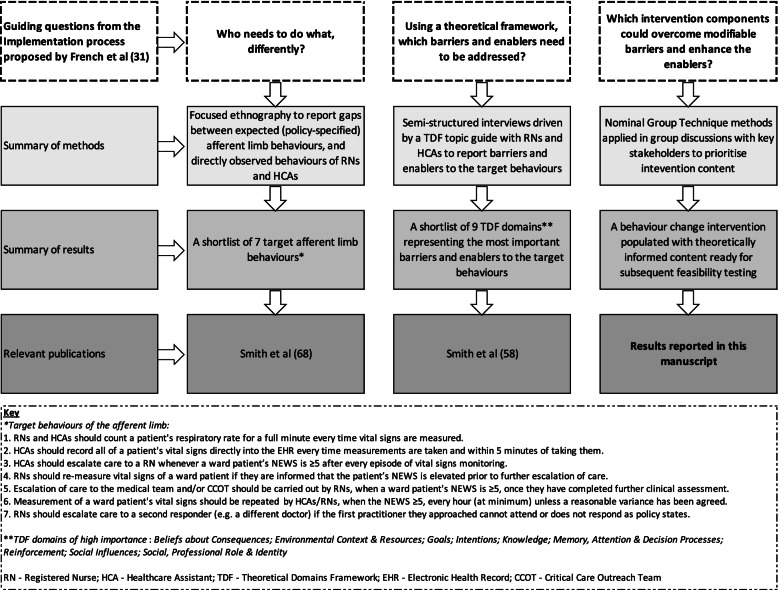
Overview of the programme of work to develop a theory-based behaviour change intervention targeting behaviours that are potential antecedents to afferent limb failure

Despite implementation of track-and-trigger tools, there is evidence that deteriorating patients continue to receive sub-optimal care [16, 17]. This has been partly attributed to ward-based nursing staff failing to recognise the abnormalities in vital signs and/or not escalating care when criteria are met [18]. This phenomenon has been termed Afferent Limb Failure (ALF) [2, 19]. ALF is increasingly reported to be associated with inconsistent behaviour of nursing staff [20, 21]. Consequently, to optimise the afferent limb and to drive more consistent responses to deteriorating patients, there is a need for interventions to support nursing staff to change their behaviour [22–24]. Theories of behaviour and behaviour change are arguably the most useful guides for developing such interventions. However, there is currently paucity of research applying behavioural theories or theoretical frameworks to explore determinants of afferent limb behaviour, or to inform selection of content for interventions to improve nursing staff’s afferent limb behaviour [25, 26]. Given evidence that systematic application of theory may increase replicability of methods [27, 28] and intervention efficacy [29, 30], the use of theory-based approaches to intervention development is justified. A multi-phase programme of work was devised modelled on the theoretically informed implementation process reported by French et al. [31] and underpinned by the Theoretical Domains Framework (TDF) (v2). A diagrammatic overview of the entire programme of work can be found in Fig. 2. In this paper, the focus is on selecting content for a behaviour change intervention.

The observable, irreducible and active elements of a behaviour change intervention that bring about the change in behaviour are termed Behaviour Change Techniques (BCTs); 93 BCTs have been identified and defined in a taxonomy [32]. The behaviour change literature distinguishes between BCTs and the strategies used to operationalise them [27]. The mechanisms through which BCTs are delivered to recipients have been labelled modes of delivery [33]. The mode of delivery may encompass the proximity of the intervention deliverer to the recipient (e.g. face-to-face, remote), the number of individuals targeted by the intervention on a single occasion (e.g. individual, dyad, group), and the medium through which BCTs are sent to intended recipients (e.g. radio, poster, mobile phone application) [32, 33]. Reporting the operational components of an intervention in sufficient detail to be replicable requires descriptions of intervention content (what); provider (who); setting (where); recipient (to whom); intensity (over how many contacts), and fidelity (the extent to which it was delivered as intended) [33]. In this work, the concrete strategies used to operationalise BCTs were labelled as *applications*. For example, social support and encouragement (*the BCT*) could be delivered face-to-face, to individual health practitioners (*mode of delivery*), through the provision of peer support workers or ‘champions’ in the workplace (*the application*).

When developing behaviour change interventions, the context in which the intervention will be delivered is recognised as an important consideration [34, 35]. It has been posited that context is both complex and multi-dimensional and extends beyond a physical space [36]. Context should be recognised as a process involving persons, resources, perspectives, and activities [37]. To design interventions feasible to deliver in practice, assessing the contextual constraints and facilitators is crucial [37]. Despite this, there is evidence of context being under-reported within the wider patient safety literature [38]. To permit suitable adjustments for context and ‘local factors’ [39] it has been recommended that interventions aiming to change health practitioners’ behaviour be developed through interactive methods with the target group, allowing local expertise and tacit contextual knowledge to be incorporated [34, 35]. The aims of this research were to select and shortlist possible BCTs, and to use structured consensus methods with healthcare staff to prioritise BCTs and applications for inclusion in a behaviour change intervention (targeting nursing staff).

## Methods

### Design

A three-stage process was used to develop the content for a theoretically informed behaviour change intervention. In stage 1, mapping tools were used to identify appropriate BCTs for the previously identified determinants of target behaviours; stage 2, using additional criteria (acceptability and feasibility) and a consensus approach, the identified BCTs were shortlisted by the research team; stage 3, shortlisted BCTs and researcher-generated applications were presented to stakeholders in online group discussions where Nominal Group Technique (NGT) methods were applied (nominal groups). To further reduce the number of applications, ranking data from nominal groups guided final consensus discussions by research team members. Permission to conduct this research was granted by a National Health Service Research Ethics Committee (REC) (reference: 18/NS/0118), the Health Research Authority (reference as for REC), and the hospital’s research and development department (reference: 18/0569).

### Mapping and shortlisting behaviour change techniques

Using linkages between TDF domains and BCTs derived from expert consensus processes [27, 40], TDF domains of high importance were mapped to specific BCTs that could be used to ameliorate barriers and/or enhance enablers associated with a given domain. A minimum of two researchers (DS and MC or JD or LMA) independently reviewed all mapped BCTs and their definitions for anticipated acceptability (to the intended recipient) and anticipated feasibility (in the intended context). For each BCT, the criteria in Table 1 were used to determine whether to include it, exclude it or bring it for discussion with all researchers (DS, MC, JD, JH, LMA). BCTs were then taken forward for discussion and voting at stakeholder groups where NGT methods were applied.

**Table 1:** Criteria applied by members of the research team during BCT shortlisting

Label applied to BCT and action	Criteria for labelling
Include – take forward for discussion at nominal groups	1. The BCT could feasibly be delivered in a clinical environment **AND** 2. The BCT is likely to be acceptable to a healthcare practitioner **AND** 3. The BCT does **not** meet exclusion criteria
Exclude – no further action	1. The BCT would take time to deliver and/or would require repeated delivery over a prolonged period (i.e. unlikely to be feasible) **AND/OR** 2. The BCT is ethically dubious e.g. applying punitive techniques to clinical staff (i.e. unlikely to be acceptable)
Uncertain – take forward for consensus discussion with the entire research team	1. Reviewer uncertain which criteria are met by the BCT – warrants further consensus discussion to inform decision-making

### Recruitment and sampling

Senior clinicians and leaders from a variety of disciplines were recruited for a leadership group and Registered Nurses (RNs) and Healthcare assistants (HCAs) from acute wards were recruited for a clinical group. These personnel were separated to reduce potential power imbalances [41]. An email outlining the nature and broad objectives of the research was sent to the chairperson/project lead of a Deteriorating Patient Steering Group (to recruit for the leadership group) and nurse managers of acute inpatient wards (to recruit for the clinical group), requesting permission to access potential participants. The project lead and ward managers then sent the invitation to potential participants via the appropriate group email. Recipients of the email were asked to contact DS if they were interested in participating. In addition, using a snowballing technique [42] any recruited participants were asked to identify colleagues from within the organisation interested in participating, and an invitation was sent to these individuals too. These approaches were repeated until an adequate sample of participants had been recruited.

### Materials

It was likely participants of the nominal groups would have no prior knowledge of behaviour change concepts and processes. Consequently, an information package (Additional file 1) was emailed to participants 2 weeks before the nominal group [43]. The information package consisted of a participant information sheet and a further document including a table showing the BCTs shortlisted in stage 2, plain-English definitions of BCTs, and example applications (minimum 1 example application per BCT). Example applications were sourced from supplementary materials accompanying the publication reporting the taxonomy of 93 BCTs [32], from educational materials developed by implementation scientists [44], and from patient safety innovations described in published literature [45, 46]. Prior to distribution, content of the information package was sense-checked by a patient advisor and by a group of clinical-academic health practitioners not directly involved in the research.

A facilitator guide was developed to structure the nominal group activities (Additional file 2). An online ranking document was also created using the Qualtrics® platform. This document included all shortlisted BCTs, and example applications presented in the information package as well as space for new suggested applications to be added during the groups. The Qualtrics® platform was selected as it permits content (i.e. new suggestions from participants) to be added in real time and to be ranked. To test the materials and the process, pilot nominal groups were held with members of an acute and critical care research group and then a health psychology research group at *City, University of London.* Facilitator guide revisions were made iteratively based on feedback from pilot group participants, and from debrief amongst research team members following piloting.

### Data collection

In the original published protocol [47], it was proposed that the groups would be conducted face-to-face. Due to the severe acute respiratory syndrome Coronavirus 2 (SARS-CoV-2) pandemic, and the consequent need to maintain social distancing and to minimise unnecessary travel [48], the groups were delivered online using Microsoft® Teams software and were facilitated by four members of the research team (DS, MC, JD, LMA).

Participants of both nominal groups were presented with an identical list of BCTs (mapped from TDF domains of high importance). After the leadership group, applications suggested by participants were incorporated as examples into the information package which was sent to participants of the subsequent (clinical) nominal group. It was anticipated that running the groups sequentially and revising the information package between groups, would enable ward nursing staff to discuss, debate and vote upon ideas proposed by senior leaders from their own organisation (alongside their own suggestions).

NGT methods involve the use of structured activities within groups comprising relevant stakeholders, with the broad aims of achieving a level of consensus and prioritising information [49]. Key activities, central to the NGT process, as described by the originators of the method are: *independent generation of ideas; ‘round-robin’ sharing of ideas; discussion and clarification of ideas, and voting (ranking of ideas)* [50]. We incorporated these key activities using a three-step process:

- Step 1: The following question was posed (by DS) to the group: ‘*Are there any other ways (or better ways) that the BCTs listed in the table could be applied in this organisation, that were not included in the information package?’* Participants silently considered the question and privately generated responses before feeding back a single idea at a time to the group. These ideas were posted onto the virtual display-board. All participants were given the chance to offer at least one idea with the exercise being repeated as many times as possible within the allotted time.
- Step 2: Participants were given the opportunity to ask questions about suggestions made by other participants and to merge suggestions considered sufficiently similar. Participants then took a short break whilst the research team met to identify any obvious discrepancies in the linkages between the BCTs and the applications suggested by participants (i.e. where the application did not reflect the BCT). Where such discrepancies were identified, a decision was made to either adjust the application to improve the alignment, propose a re-alignment of the application to a more suitable BCT from the shortlist, or exclude the application. The decision to exclude was made when the suggested application did not align with any of the BCTs and/or did not target the previously identified barriers/enablers. These decisions were driven by health psychologists (MC, JD) within the research team*.* Following any adjustments, new applications (i.e. those suggested by the group) from the virtual display-board were added onto the online ranking documents.
- Step 3: The health psychologists summarised to the participants any adjustments that had been made during the break time and offered them the opportunity to comment. A hyperlink was then posted into the discussion thread so that participants could access the ranking document in Qualtrics®. From the longer list provided, participants were asked to rank the five BCTs/applications that they considered would be most acceptable [51] to ward staff from 1 (most acceptable) to 5 (least acceptable). Participants were then requested to repeat this activity according to how feasible it would be to deliver the BCTs/applications.

### Data analysis

Scores were assigned to each of the BCTs/applications based on the ranking information from participants [52]. Where a BCT/application was ranked first by a participant it was scored 5; second it was scored 4; third it was scored 3 etc. Participants’ scores were summed to identify ranked priorities from within and across the two nominal groups [52]. For example, if 12 participants voted for any single BCT/application then the maximum score was 60 (i.e. 12 × 5, requiring all participants to rank the item first). In contrast, if a BCT/application was not ranked by any participants it would score 0. Summed scores and percentages were calculated. The frequency that each BCT/application was prioritised by a participant (i.e. ranked 1–5) was also counted for both ranking activities i.e. acceptability and feasibility.

All combinations of BCTs/applications were reviewed during subsequent consensus discussions involving nurse academics (DS, LMA), health psychology academics (MC, JD), and a lead nurse (JH). Where a single BCT had several potential applications, nominal group ranking data were used to prioritise which specific application/s to include in the intervention (higher scoring and more frequently prioritised applications were included). Where a BCT/application combination received a low score from nominal groups, and/or was not frequently prioritised (i.e. not frequently ranked 1–5), the decision to include or exclude from the intervention was made through discussion and debate, guided by the following considerations:

- The potential consequences of eliminating the BCT and its application/s on the theoretical integrity of the intervention (i.e. where exclusion would result in specified TDF domain/s and/or target behaviours not being addressed by intervention content).
- Further scrutiny of the BCT and its application/s in relation to the APEASE criteria (where APEASE stands for acceptability, practicability, effectiveness, affordability, side effects, equity) [33]. We found that applying the APEASE criteria at this stage in the consensus process (i.e. when BCTs were being scrutinised alongside potential applications) allowed us to apply all criteria to some extent. We contend this may not have been possible had we applied APEASE before BCTs had been linked to specific applications. To exemplify, we were able to judge the potential ‘affordability’ of the BCT *Prompts/cues* more accurately once we had clarity that the BCT would be delivered using a simple laminated sign (a relatively inexpensive mechanism in this context).

## Results

We recruited 31 participants in total for the nominal groups. Six individuals withdrew on the day of the group and 6 did not attend. Twelve participants attended the leadership group (NGT1), and 7 participants attended the clinical group (NGT2) (the professional roles of participants are displayed in Additional file 3).

The mapping exercise (stage 1) resulted in a provisional list of 50 unique BCTs (listed in Additional file 4). From the application of shortlisting criteria (Table 1) and consensus discussions within the research team (stage 2), 38 BCTs were excluded resulting in a shortlist of 14 unique BCTs for discussion and prioritisation at the nominal groups (stage 3).

**Fig. 3:**
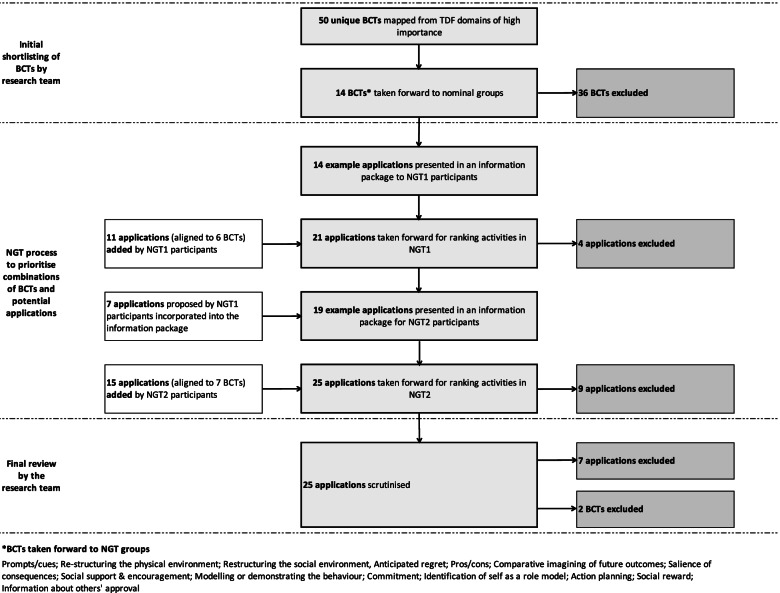
A summary of BCT shortlisting process, and the numbers of applications added and excluded across the consensus process

The duration of both nominal groups was 2 hours. Across the groups, 24 new applications were proposed for applying the BCTs. Eleven of the applications proposed by participants were considered appropriate for one or more of the 14 shortlisted BCTs. The number of applications added and excluded at different stages of the NGT process is summarised in Fig. 3. In NGT 1, 11 online Qualtrics® ranking forms were completed for the first ranking task (acceptability of different BCT and application combinations) whilst 13 forms were completed for the second ranking task (feasibility of different BCT and application combinations). This discrepancy implies that one participant did not complete the acceptability ranking document but instead completed the feasibility document twice. As both ranking documents included the same content (only the heading and explanatory text varied), the summative scores were unlikely to be affected. In NGT 2, 6 ranking forms were completed for ranking task 1; with 7 completed for ranking task 2 implying that 1 participant did not rank for acceptability. This explains the variation in the denominator for the summative scores. A detailed breakdown of ranking data for both nominal groups can be found in Table 2.

**Table 2:** Total scores from both ranking tasks (*A* - acceptability and *F* - feasibility) across the two nominal groups, and the decision to include or exclude the BCT/application from the intervention with brief rationale

No.	Behaviour Change Technique (BCT)	Application (concrete strategy for delivering the BCT in practice)	Total scores from ranking activities (% scores)				Total score	Frequency BCT/application ranked top 5 (denominator)		Included in the intervention		Brief rationale for decision to include or exclude from the intervention
			NGT1		NGT2			NGT1 (24)	NGT2 (13)			
			A (% of 55)	F (% of 65)	A (% of 30)	F (% of 35)				Yes	No	
1a	Prompts/cues	Attach laminated signs to vital signs monitoring equipment to prompt the desired behaviour +	10 (16)	46 (71)	0 (0)	14 (40)	70	12	3	✓		Highest scoring application for this BCT from nominal group participants
1b	Prompts/cues	Use best practice advisory ‘pop-ups’ on the Electronic Health Record to prompt the desired behaviour ∆	9 (16)	3 (5)	0 (0)	6 (17)	18	6	2		✓	Alternative (1a) application of this BCT favoured by nominal group participants
2a	Re-structuring the physical environment	Add vital signs monitoring equipment to the environment +	34 (62)	20 (31)	9 (30)	0 (0)	63	14	2	✓		High scoring from nominal group/s
2b	Re-structuring the physical environment	Add a visual marker on the floor to signal where the vital signs monitoring equipment should stand ∆	13 (24)	30 (46)	N/A	N/A	43	13	N/A	✓		High scoring from nominal group/s
2c	Re-structuring the physical environment	Add clocks with second hands to the ward to enable monitoring of respiratory rate ∆	2 (4)	15 (23)	3 (10)	11 (31)	31	7	3	✓		High scoring from nominal group/s
2d	Re-structuring the physical environment	Add more digital thermometers with timers for 15 s, 30s, 60s etc. to enable monitoring of respiratory rate ∅	N/A	N/A	3 (10)	9 (26)	12	N/A	4	✓		To ensure coverage of all target behaviours
3	Anticipated regret	*Workshop based* – ask RN/HCA to consider the degree of regret that they might feel if the desired behaviour was not enacted, and a patient came to harm +	4 (7)	0 (0)	4 (13)	0 (0)	8	1	1	✓		To ensure coverage of all TDF domains of high importance and all target behaviours
4	Pros/cons	*Workshop based* – ask RN/HCA to list and compare pros and cons of enacting the desired behaviour +	8 (15)	1 (2)	0 (0)	0 (0)	9	3	0	✓		To ensure coverage of all TDF domains of high importance and all target behaviours
5a	Re-structuring the social environment	Set the expectation that HCAs attend ward safety huddles alongside RNs to facilitate escalation of deteriorating patients +	7 (13)	3 (5)	5 (17)	3 (9)	18	3	2	✓		Highest scoring application for this BCT from nominal group participants
5b	Re-structuring the social environment	Proactively roster HCAs who will attend the safety huddles ∆	9 (16)	4 (6)	N/A	N/A	13	4	N/A		✓	Decision made that all HCAs should be encouraged to attend the safety huddle
5c	Re-structuring the social environment	Formalise a ‘HCA in-charge role’ and ensure clear expectations/training ∅	N/A	N/A	8 (27)	1 (3)	9	N/A	3		✓	Alternative (5a) application of this BCT favoured by nominal group participants
5d	Re-structuring the social environment	Use clinical cases during safety huddles as a stimulus for conversation ∆	8 (15)	0 (0)	0 (0)	9 (26)	17	3	2	✓		High scoring from nominal group/s
6	Comparative imagining of future outcomes	*Workshop based* – prompt HCAs to imagine and compare likely or possible outcomes following immediate escalation of a deteriorating patient to the RN versus no escalation or delayed escalation +	2 (4)	4 (6)	2 (7)	0 (0)	8	2	1	✓		To ensure coverage of all TDF domains of high importance and all target behaviours
7a	Salience of consequences	*Workshop based* – show videos of patients speaking emotively about the consequences of delayed escalation and timely escalation +	7 (13)	0 (0)	11 (37)	5 (14)	23	3	4	✓		High scoring from nominal group/s
7b	Salience of consequences	*Workshop based* – show videos of patients speaking emotively about the consequences of timely escalation ∅	N/A	N/A	11 (37)	5 (14)	16	N/A	6	✓		Could be easily delivered alongside 7a
8a	Social support and encouragement	Deploy deteriorating patient champions (HCA and RN level) and ensure clear expectations/training +	8 (15)	4 (6)	8 (27)	17 (49)	37	6	9	✓		Highest scoring application for this BCT from nominal group participants
8b	Social support and encouragement	Allocate junior HCAs a senior HCA mentor ∆	11 (20)	4 (6)	3 (10)	0 (0)	18	7	1		✓	Alternative (8a) application of this BCT favoured by nominal group participants
9a	Modelling or demonstrating	*Workshop based* – provide a video of a senior and respected staff member enacting the desired behaviour/s +	6 (11)	4 (6)	1 (3)	4 (11)	15	4	3		✓	The relevant target behaviours would be difficult to model using this application i.e. not practical.
9b	Modelling or demonstrating	Senior nurse/s return to clinical practice and model the desired behaviours e.g., monitoring the vital signs and using NEWS appropriately ∆	10 (18)	10 (15)	2 (6)	5 (14)	27	5	3		✓	Unlikely to be sustained (not practical), may be expensive and could have negative side effects.
10	Commitment	*Workshop based* – use “I will” statements to affirm an intention e.g., the intention to monitor respiratory rate with every set of vital signs +	2 (3)	0 (0)	0 (0)	0 (0)	2	1	0		✓	May be viewed by clinical staff as patronising – difficult to deliver in a meaningful way
11	Identification of self as a role model	*Workshop based* –ask RNs to picture themselves enacting the desired behaviour/s and then ask them to consider who might be learning from their good practice +	2 (3)	0 (0)	0 (0)	0 (0)	2	1	0	✓		To ensure coverage of all TDF domains of high importance and all target behaviours
12	Action planning	*Workshop based* - develop “if … then” statements to link a cue to the desired behaviour +	3 (5)	3 (5)	0 (0)	0 (0)	6	1	0	✓		To ensure coverage of all TDF domains of high importance and all target behaviours
13	Social reward	Senior staff on the ward praise junior staff when they enact the desired behaviour +	4 (7)	7 (11)	2 (6)	2 (6)	15	5	3	✓		To ensure coverage of all TDF domains of high importance and all target behaviours
14a	Information about others’ approval	*Workshop based* – show a video of senior and credible nursing staff describing the behaviours that they approve of +	0 (0)	2 (3)	5 (17)	0 (0)	7	1	2	✓		To ensure coverage of all TDF domains of high importance and all target behaviours
14b	Information about others’ approval	CCOT nurses to provide feedback to ward staff on their approval of appropriate escalation. Feedback should be given as soon after the escalation event as possible ∅	N/A	N/A	11 (36)	14 (40)	25	N/A	9	✓		Highest scoring application for this BCT from nominal group participants

Key:

A = Total score from ranking activity related to the perceived acceptability of the BCT/application combination to ward nursing staff

F = Total score from ranking activity related to the perceived feasibility of the BCT/application combination to ward nursing staff

+ Application from the information pack compiled by the research team

∆ Application proposed during NGT1 (the leadership group)

∅ Application proposed during NGT2 (the clinical group)

*HCA* Healthcare assistant, *RN* Registered Nurse, *CCOT* Critical Care Outreach Team

**Fig. 4:**
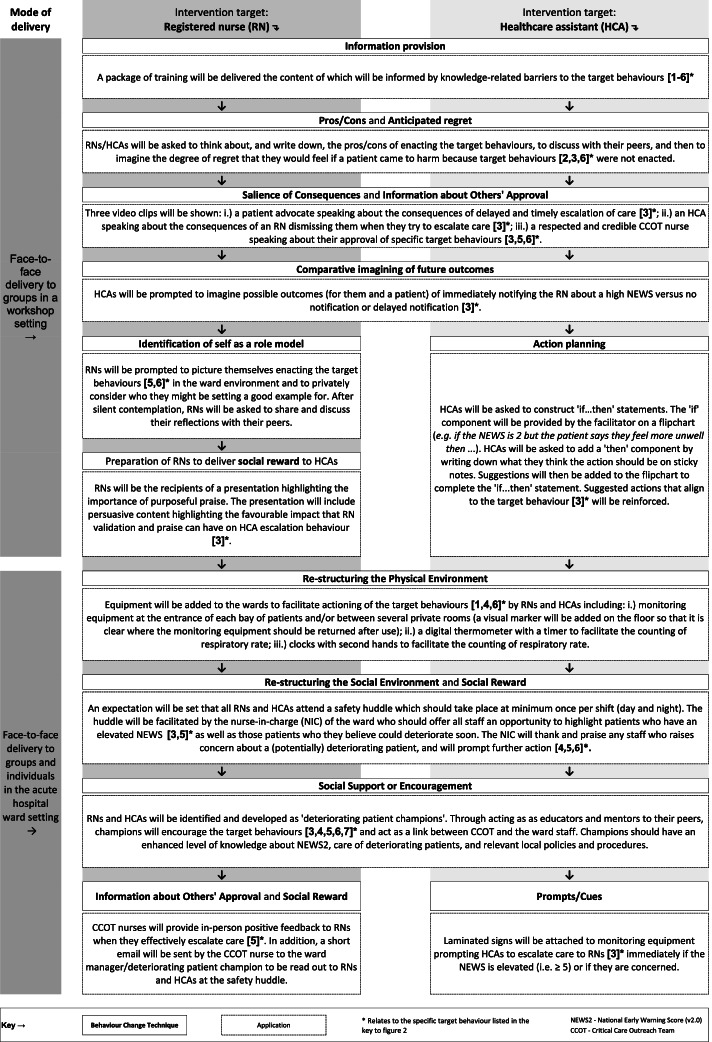
An overview of a theoretically informed behaviour change intervention to drive more consistent behaviours of the afferent limb of the rapid response system by nursing staff

The intervention (summarised in Fig. 4) was populated with 12 BCTs that will all be delivered face-to-face at group and individual levels (the modes of delivery), through 18 different applications. Four BCTs (*Re-structuring the physical environment, Re-structuring the social environment, Salience of consequences, Information about others’ approval)* will be delivered using multiple applications. A brief rationale for decisions made during consensus discussions regarding which BCTs/applications were included and excluded from the intervention is provided is Table 2.

## Discussion

Fifty BCTs (mapped from nine domains of the Theoretical Domains Framework) that could be used to change behaviour of RNs and HCAs were shortlisted to 14 and, alongside example applications, presented to key stakeholders in two virtual nominal groups. Participants proposed 11 new applications for the BCTs and ranked BCTs/applications (including examples provided by the research team and those suggested by nominal group participants) for acceptability to nursing staff and feasibility for delivery in an acute hospital ward. Ordinal data from ranking tasks were used to inform content of the intervention which has been populated with 12 BCTs, that will be delivered through 18 different applications in either a workshop or ward setting.

Whilst the TDF has been widely used to report barriers and enablers to health behaviour change with patients, its application in the design of interventions targeting healthcare practitioners is more limited. A systematic review was conducted to synthesise international literature reporting application of the TDF in designing interventions to support healthcare practitioner behaviour change [53]. The authors reported that only around 20% of articles (i.e. 60/297) reporting use of the TDF to explore implementation problems, extended its use to intervention design [53]. In recently updated guidelines from the Medical Research Council [36], methodological innovation and the adoption of new methods are highlighted as important for the future development of intervention research. We contend the use of NGT methods provides a structured, replicable, and expedient approach for ideas sharing and consensus building when designing a behaviour change intervention.

The interaction of an intervention with context is a crucial consideration for researchers spanning the phases of intervention design, evaluation, and implementation [34–36]. The impact of an intervention may be increased when its components are adjusted to best suit the context within which it is being delivered (i.e. when the intervention is tailored to a specific group or a particular setting) [36, 54–56]. To ensure the theoretical basis of the intervention is not compromised, it is advocated researchers reach agreement about the degree of variation that is permissible and prohibited, i.e. which components of an intervention can be adjusted and which must be maintained [36, 57]. To ensure the theoretical integrity of the intervention was upheld during NGT activities, we presented participants of both groups with an identical list of BCTs and applications and explained that the BCTs were ‘fixed’, but the applications could be revised or elaborated. We suggest our reported methods could be replicated in different settings, and with different stakeholders, to determine how specified BCTs could be operationalised in different contexts and tailored for different groups.

There was overlap in the TDF domains that represented important barriers and enablers to the target behaviours for both RNs and HCAs [58]. Similar overlap in the determinants of behaviour change, between different healthcare practitioners, has been reported in other work [59]. This overlap explains why the majority of BCTs included in our intervention will be directed at both RNs and HCAs. From our list of target behaviours (see the key in Fig. 2), three are enacted by RNs, two are enacted by HCAs, and two are enacted by RNs *and* HCAs. This implies that some target behaviours are enacted by individuals occupying a specific role (i.e. RN *or* HCA), whilst for others responsibility for enactment is shared. The individual responsible for enacting a specific behaviour has been termed ‘the actor’ [60]. Clearly specifying each target behaviour, including the actor/s, enabled us to evaluate the suitability of each application for the intended recipient/s and, where necessary, to tailor the application accordingly. For example, the laminated signage (used to apply the *Prompts/cues* BCT) will incorporate a tailored message directed specifically towards HCAs.

Our intervention includes some BCTs and applications where the mode of delivery will be a face-to-face workshop, and some for delivery in the clinical setting (ward-based applications). The ranking information from the nominal groups suggests stakeholders broadly perceived ward-based applications to be more acceptable and feasible than workshop-based applications. To attend workshops, staff must be released from their usual clinical duties. In several studies, different healthcare practitioners have reported a lack of time and/or short staffing as barriers to participation in various activities [61, 62]. This may explain why workshop-based applications were viewed less favourably by participants. Where the application of a BCT involved modifying an existing patient safety mechanism ranking scores were favourable. An example of this is the application of the BCT *Re-structuring the Social Environment* through the re-organisation of ‘safety huddles’ (brief discussions that take place during a shift, between groups of clinical staff, with a focus on patient safety [45]). It is plausible that adjusting existing practices was perceived by participants to be less arduous than introducing new approaches. Notwithstanding the potential challenges of delivering BCTs through workshops, we retained this mode of delivery for several applications, adopting a similar combined approach as reported in other published work [63]. When working in the clinical setting, healthcare practitioners often experience high cognitive load associated with interruptions and distractions [64, 65]. On this basis, we contend that some BCTs would be best applied outside the clinical environment, particularly where the specific applications involve participants imagining different clinical scenarios and/or reflecting on clinical practice. However, the acceptability and feasibility of delivering this combined intervention in the ‘real world’ setting will need to be explored further through piloting [36].

In a previous publication from this programme of work [58], the TDF domain *Knowledge* was identified as representing important barriers and enablers to the target behaviours. Despite this, none of the specific BCTs mapped from this domain were considered suitable for inclusion in this intervention. Whilst educational approaches alone are unlikely to be sufficient to drive behaviour change [66, 67], possession of knowledge is often a pre-requisite to the decisions individuals make and the behaviours they enact [67]. Consequently, despite the lack of appropriate BCTs, we opted to include a training component to our intervention that will address specific knowledge-related barriers identified from earlier empirical work [58, 68]. The importance of this is underscored by the wider literature where knowledge deficits have been reported as antecedents to afferent limb failure [21, 69, 70].

Throughout the process, we iteratively reviewed the broader dataset to ensure alignment between target behaviours, TDF domains, BCT/s, and their suggested application/s (this occurred during BCT shortlisting, rapidly during nominal groups, and more deliberatively during final consensus discussions). The importance of having continual oversight of the broader corpus of data to inform decision-making is highlighted by our handling of the BCT *Commitment.* This was the only shortlisted BCT linked to the TDF domain *Intentions* (a domain of high importance). Results of TDF-driven interviews (carried out earlier in this programme of work), confirmed that participant beliefs within this domain reflected strong intention to enact target behaviour/s (i.e. beliefs were enabling) with no modifiable barriers identified [58]. Consequently, inclusion of the BCT *Commitment*, which has the purpose of strengthening intention to change behaviour [32], was deemed redundant. Using findings of empirical work to inform pragmatic decision-making in this way enabled us to keep the number of BCTs to a minimum, which should increase the likelihood the intervention can be delivered to RNs and HCAs with high fidelity [59, 71].

### Limitations

At present, there is no clear evidence base demonstrating that certain BCTs are more effective than others in relation to specific TDF domains. Consequently, we were reliant on expert consensus literature to identify BCTs that could be used to populate the intervention. The work by Cane et al. [40] (our primary source for BCT mapping) did not yield BCTs for two of our domains of high importance (*Memory, Attention and Decision Processes* and *Social, Professional Role and Identity*). Consequently, we relied on the original mapping matrix by Michie et al. [27] to identify additional techniques suitable for these domains. Whilst there is precedent for using these two reference sources in combination [59, 72], there is currently no single best approach for mapping TDF domains to BCTs.

Approximately 40% of individuals who volunteered to participate withdrew and/or did not attend their allocated nominal group. This resulted in a smaller than anticipated number of participants despite our decision to over-recruit. It is plausible that increased pressure on healthcare staff from the Coronavirus pandemic contributed to participant withdrawal, particularly as our clinical group participants were nursing staff involved in delivering direct patient care. Despite a smaller than anticipated number of participants, the clinical group included representatives from all grades of nursing staff who will potentially receive the intervention.

Only one HCA attended the clinical group. As HCAs are intended recipients of the intervention, the lack of representation is a noteworthy limitation. Given the potential importance of intervention acceptability in determining uptake of an intervention in practice [73], it has been advocated that intervention acceptability be assessed during feasibility testing [36]. We plan to use the Theoretical Framework of Acceptability [73] during feasibility testing to further examine the acceptability of our proposed intervention to HCAs (and other key stakeholders).

The information package provided to participants ahead of the nominal groups included a list of BCTs, their definitions, and example applications for each BCT. Providing example applications may have induced cognitive bias and specifically ‘anchoring’ [74]. That is, participants may have given a disproportionate level of thought to the example applications provided rather than considering alternate means of operationalising BCTs [74]. We attempted to mitigate this by emphasising the applications were only examples and through repeated encouragement of participants to think creatively and to share their own ideas. Notwithstanding this limitation, given our participants were healthcare staff who were largely naïve to behaviour change methods, it is unlikely we would have completed all stages of the process, in the time available, if materials had not been provided beforehand [75, 76].

## Conclusions

In this paper we present a behaviour change intervention populated with 12 theoretically informed BCTs that could be translated into practice through 18 different applications. Decision making regarding the content of the intervention was driven by information from group discussions where nominal group technique methods were applied. To the best of our knowledge, this is the first report of NGT methods being used to shape the content of a theory-based behaviour change intervention aimed at strengthening the afferent limb of the rapid response system. Further work will involve feasibility testing and expanding the detail of reporting (to the level of an intervention manual) to permit potential replication and evaluation.

## Supplementary Information

**Additional file 1.** Information package for nominal group participants.

**Additional file 2.** Facilitator’s guide for nominal groups.

**Additional file 3.** Professional role of participants attending nominal groups.

**Additional file 4.** The number and labels of the Behaviour Change Techniques (BCTs) mapped from each of the 9 Theoretical Domains Framework (TDF) domains of high importance.

## Data Availability

All data generated or analysed during this study are included in this published article [and its supplementary information files].

## Abbreviations

ALF: Afferent Limb Failure
APEASE: Acceptability, Practicability, Effectiveness, Affordability, Side effects, Equity
BCT: Behaviour Change Technique
ICU: Intensive Care Unit
NEWS: National Early Warning Score
NGT: Nominal Group Technique
RRS: Rapid Response System
SAE: Serious Adverse Event
TDF: Theoretical Domains Framework
UK: United Kingdom
