# Supplementary material for: Selecting intervention content to target barriers and enablers of recognition and response to deteriorating patients: an online nominal group study
Source: BMC Health Serv Res. 2022 Jun 10;22:766. doi: 10.1186/s12913-022-08128-6 (PMC9186287; doi:10.1186/s12913-022-08128-6)
Supplement: Supplementary file 1 — Additional file 1. Information package for nominal group participants. [file 12913_2022_8128_MOESM1_ESM.pdf]

DEveloping a Complex Intervention for DEteriorating Patients using Theoretical Modelling (DECIDE study)

Information package for participants (phase 3)

What is the purpose of this document?

Thank you for expressing interest in participating in a group discussion using nominal group technique. By now, you should have received a participant information sheet (v3.0 20/7/20) with more detailed information about the research. Please read this sheet before you read this package. This document should complement the information in the information sheet and provide you with more detail about what you will be discussing at the meeting.

What research has already been completed?

In phases 1 and 2 of this research project, data were collected by observing the behaviour of nursing staff when monitoring patients' clinical observations and responding to signs of deterioration. Nursing staff (Registered Nurses (RNs) and Healthcare Assistants (HCAs)) were then interviewed to explore their views on what influences their behaviour in response to patient deterioration. These data have now been analysed, using structured approaches, and a theoretical framework of behaviour change applied.

From these earlier processes, the research team have put together a list of **Behaviour Change Techniques (BCTs)** that respond to the barriers to ideal practice that nursing staff describe. The BCTs are linked to behavioural theories and could change staff behaviour and improve responses to deteriorating patients. BCTs are considered the 'active ingredients' of behaviour change interventions and broadly work by either promoting desired behaviours or inhibiting unwanted behaviours.

Multiple BCTs are available to address the identified barriers and enablers, but it is likely that some of them will be easier to deliver and more acceptable to staff in the Trust than others. The group process that you have been invited to will help to identify the optimal combination of techniques that are both acceptable and feasible.

What will we be discussing?

During the group discussion you will be asked to consider the BCTs listed in table 1 over the page and, specifically, how they could be applied on the wards in your Trust.

To help put these techniques into context, we have offered some examples of the barriers (i.e. factors that prevent a RN/HCA doing the right thing) and enablers (i.e. factors that help a RN/HCA to do the right thing) that staff who were interviewed believed affected their behaviour when monitoring patients' clinical observations and escalating care. To help you to make sense of the BCTs, descriptions in plain-English are included in the table on the next page. You will also find some examples of how these BCTs could be applied in your Trust (these are just examples; you may have alternative suggestions which you will be invited to share at the group meeting).

ACTION POINT: It would be helpful if you could read through the list of techniques and example applications before attending the group. It will also be very helpful if you can see the table of examples during the meeting so that you can refer to it. As such, you may wish to print the table out before the meeting and have it with you or, alternatively, have it visible on a second computer screen or different device during the group discussion. There will be an opportunity at the start of the group to ask questions about any of the BCTs that you have not understood and/or would like more information about.

Row no.	**Example** beliefs from interview participants reflecting barriers and enablers	Behaviour Change Technique/s (BCT)	Plain-English explanation of the BCT	**Examples** of how the BCT/s could be delivered in your Trust (the application)
1a	If a patient has abnormal vital signs/an elevated NEWS, some HCAs attempt to improve the score with interventions (e.g. asking the patient to drink more) or perform further monitoring rather than escalating immediately to a RN (potential barrier).	Prompts/cues	Introduce an item into the environment that will prompt or cue the behaviour. The prompt or cue would normally occur at the time or place that the behaviour is performed.	**Could be delivered in the ward setting:** Laminated signs are attached to the DINAMAP reminding HCAs that they should immediately escalate a NEWS of 5 or more to a RN so that they can assess the patient further.
1b	Staff believe that the nurse-in-charge of the ward and/or senior nursing colleagues are resources to be called upon when a patient deteriorates (potential enabler).	Prompts/cues	Introduce an item into the environment that will prompt or cue the behaviour. The prompt or cue would normally occur at the time or place that the behaviour is performed.	**Could be delivered in the ward setting:** A pop-up or best practice advisory is incorporated into EPIC to prompt the RN to notify the nurse-in-charge about a deteriorating patient.
2a	The monitoring of clinical observations may be hindered by a lack of physical resources, in particular a lack of DINAMAPS and digital thermometers (potential barrier).	Re-structuring the physical environment	Change the physical environment to facilitate performance of the wanted behaviour or create barriers to the unwanted behaviour.	**Could be delivered in the ward setting:** DINAMAPS and digital thermometers are added to the ward environment and positioned close to the entrance of each bay so that they are easily accessible to RNs/HCAs. A marker is added to the floor so it is clear where the equipment should be returned to.
2b		Re-structuring the physical environment	Change the physical environment to facilitate performance of the wanted behaviour or create barriers to the unwanted behaviour.	**Could be delivered in the ward setting:** Wall mounted clocks with a second hand are added to the ward environment to facilitate the accurate measurement of respiratory rate.
3	HCAs believe that patients may be upset if they are woken overnight to have their vital signs monitored (potential barrier).	Anticipated regret	Create an awareness of the future regret that will be felt if the unwanted behaviour is performed.	**Could be delivered in a deteriorating patient workshop:** Staff are asked to think about the degree of regret that they might feel if a patient came to harm because their condition worsened, and was not detected quickly, because vital signs were not monitored overnight.
4		Pros/Cons	Prompt people to identify and compare reasons for wanting (pros) and not wanting to (cons) change their behaviour.	**Could be delivered in a deteriorating patient workshop:** Advise RNs/HCAs to list and compare the advantages and disadvantages of waking patients up to perform vital signs monitoring overnight.

Row no.	**Example** beliefs from interview participants reflecting barriers and enablers	Behaviour Change Technique/s (BCT)	Plain-English explanation of the BCT	**Examples** of how the BCT/s could be delivered in your Trust (the application)
5a	Some HCAs believe that they are not able to regularly attend ward huddles or do not find them a useful resource for drawing attention to deteriorating patients (potential barrier).	Re-structuring the social environment	Change the social environment to facilitate performance of the wanted behaviour or create barriers to the unwanted behaviour.	**Could be delivered in the ward setting:** Set the expectation that at least one HCA representative per shift will attend the ward safety huddles. Plan, ahead of time, which HCA/s will attend the huddles alongside registered colleagues. This could be included on the staff duty rota. Prior to attending the safety huddle, the senior HCA would be asked to check in with all their HCA colleagues on duty and ask the following questions: "do any of your patients have an elevated NEWS?" and/or "are you worried that any of your patients are deteriorating or likely to deteriorate?". These concerns would be escalated to RNs (including the nurse-in-charge) at the huddle.
5b		Re-structuring the social environment	Change the social environment to facilitate performance of the wanted behaviour or create barriers to the unwanted behaviour.	**Could be delivered in the ward setting:** Incorporate short case study discussions into safety huddles. Encourage HCAs to present a case study and talk about their role in the care of a patient who was deteriorating or vulnerable to deterioration.
6	Some HCAs believe that when they escalate to a RN their concerns will be dismissed, or the RN will 'explain away' the elevated NEWS (potential barrier).	Comparative imagining of future outcomes	Prompt people to imagine and compare future outcomes of changed versus unchanged behaviour.	**Could be delivered in a deteriorating patient workshop:** Prompt HCAs to imagine and compare likely or possible outcomes following immediate escalation of an elevated NEWS to the RN versus no escalation or delayed escalation.
7		Salience of consequences	Emphasise the consequences of performing/not performing the behaviour with the aim of making them more memorable.	**Could be delivered in a deteriorating patient workshop:** Provide a short video clip of a patient talking emotively about the negative consequences that delayed escalation (when they deteriorated) had on their future health and wellbeing. Provide an alternate video with a different patient talking about the positive consequences that timely escalation had on their future health and wellbeing.

Row no.	**Example** beliefs from interview participants reflecting barriers and enablers	Behaviour Change Technique/s (BCT)	Plain-English explanation of the BCT	**Examples** of how the BCT/s could be delivered in your Trust (the application)
8a	Experienced HCAs believe that it is their role to teach or "prompt" new HCAs on how to use the monitoring equipment and/or how to record vital signs (potential enabler).	Social support or encouragement	Advise on, arrange, or provide social support, praise, or reward for performance of the behaviour.	**Could be delivered in the ward setting:** From the existing ward team, identify local deteriorating patient champions (at both RN and HCA level). These champions could provide ward-based support and encouragement to their colleagues to enact 'best practice' behaviours when monitoring and recording vital signs.
8b	Experienced HCAs believe that it is their role to teach or "prompt" new HCAs on how to use the monitoring equipment and/or how to record vital signs (potential enabler).	Social support or encouragement	Advise on, arrange, or provide social support, praise, or reward for performance of the behaviour.	**Could be delivered in the ward setting:** From the existing ward team, allocate new/junior HCAs a more senior 'HCA mentor' who will support and encourage them to deliver best practice behaviours when monitoring and recording vital signs.
9a	Staff believe that their colleagues have/do not have a positive and encouraging attitude towards them when they are monitoring vital signs and escalating deterioration (potential barrier).	Modelling or demonstrating the behaviour	Provide a visual sample of the behaviour being performed. This could be directly in person or indirectly (e.g. via film, pictures) for the person to work towards.	**Could be delivered in a workshop setting:** RNs/HCAs are shown a short video clip of a respected and credible senior CCOT[1] nurse modelling the monitoring of vital signs (including the manual measurement of respiratory rate) and escalating care using the ISBARD[2] communication tool.
9b	Staff believe that nursing colleagues perceived to be 'senior' and 'experienced' positively influence their behaviour when measuring vital signs and escalating deterioration (potential enabler).	Modelling or demonstrating the behaviour	Provide a visual sample of the behaviour being performed. This could be directly in person or indirectly (e.g. via film, pictures) for the person to work towards.	**Could be delivered in the ward setting:** Senior nurses and matrons intermittently return to the floor and participate in clinical assessment of patients including the monitoring of vital signs to role model good practice for junior RNs and HCAs.
10	HCAs intend to monitor patient's respiratory rates when measuring vital signs (potential enabler).	Commitment	Ask the person to make a statement indicating a commitment to change behaviour.	**Could be delivered in a deteriorating patient workshop:** Ask staff to make a commitment using an "I will" statement. Here, the "I will" statement will relate to the intention to monitor respiratory rate every time vital signs are measured. This statement could be recorded on a sticky note or a postcard and returned to the individual a month or so later to remind them of their commitment.

Row no.	**Example** beliefs from interview participants reflecting barriers and enablers	Behaviour Change Technique/s (BCT)	Plain-English explanation of the BCT	**Examples** of how the BCT/s could be delivered in your Trust (the application)
11	HCAs believe that the frequency of vital signs are measured is influenced by instructions from the RN (potential enabler).	Identification of self as a role model	Inform people that their behaviour may be an example to others.	**Delivered in a deteriorating patient workshop:** After discussing the circumstances in which monitoring of vital signs should be increased, RNs are asked to picture themselves explicitly delegating repeat monitoring to an HCA. RNs are then asked to identify who might be learning from their good practice.
12	If the medical team responsible for a patient who is deteriorating do not respond when called, RNs might reach-out to other potential responders for assistance including other medical staff on the ward and/or CCOT[1] (potential enabler).	Action planning	Prompt detailed planning of performance of the behaviour (must include at least one of the following: when, where, how often, and for how long, the behaviour should be performed).	**Could be delivered in a deteriorating patient workshop:** Ask RNs to think of cues that help them to escalate a deteriorating patient appropriately to different responders. Request that RNs produce "if…then" statements linking a cue to the correct behaviour. This could be carried out with sticky notes on a board e.g. ask the group to use sticky notes to record cues ("if") and then to repeat the exercise with actions on new sticky notes ("then"), before linking the cues and behaviours together.
13	Staff believe that when they demonstrate good practice in escalating a deteriorating patient, their behaviour may be reinforced with positive feedback from another member of nursing staff or a doctor (potential enabler).	Social reward	Arrange verbal or non-verbal reward if there has been effort and/or progress in performing the behaviour includes Positive reinforcement.	**Could be delivered in the ward setting:** Senior RNs (e.g. ward managers, nurse in charge, CPFs, deteriorating patient champions) to thank and praise staff whenever they escalate an elevated NEWS appropriately.
14	RNs tendency to escalate care for a deteriorating patient is influenced by the response that they get from the CCOT[1] nurse (positive or negative) (potential barrier or enabler).	Information about others' approval	Provide information about what other people think about the behaviour. The information clarifies whether others will like, approve, or disapprove of what the person is doing or will do.	**Could be delivered in a deteriorating patient workshop:** RNs are shown a short video clip of senior CCOT[1] nurses describing the behaviours that they approve of in relation to escalation of care for a deteriorating patient.

[1] Critical Care Outreach Team

[2] **I**ntroduction, **S**ituation, **B**ackground, **A**ssessment, **R**ecommendation, **D**ecision - a structured communication tool used within the Trust to facilitate clinical conversations specifically related to escalation of care for a deteriorating patient.

How will I access the group given the current COVID-19 pandemic?

Considering the COVID-19 pandemic, the group discussion will be carried out online using Microsoft (MS) Teams. In appendix 1, you will find additional information on how to access the online group, some basic ground rules when participating in an online meeting, and practical information about how to use MS Teams during the meeting.

ACTION POINT: Even if you are familiar with MS Teams, please try to read the ground rules section (section 2) and the participation section (section 3) beforehand, as this may be helpful.

To participate, you will need access to a computer, laptop, tablet or, at minimum, a smart phone with internet access. Your device will need a working microphone and, ideally, a functioning video camera. To maintain connectivity, you will need to be logged onto a stable internet connection during the group. Whilst we do not anticipate very sensitive or confidential content arising during the discussion, it would still be advisable to try and position yourself in a quiet and private environment during the group.

Before the group, the researcher will offer you the chance to do a test call on MS Teams. This will involve a short call (likely 5-10mins) with the researcher, where you both login to MS Teams together to check that your connection is satisfactory and that your microphone and, if appropriate, video camera is working too. A test call is not compulsory but may be useful particularly if you have not used MS Teams before or are unsure about the effectiveness of your IT equipment.

What if my situation changes on the day of the group and I cannot attend?

If you cannot attend, please feel free to email the researcher [redacted] to notify him beforehand. This is not mandatory but will help him to know not to expect you in the group.

What if I am held up and cannot join the group on time?

Access to the group will close 10 minutes after the start time. Unfortunately, this means that if you are more than 10 minutes late you will not be able to join. This is to prevent disruption for other participants.

What if too few people turn up to the group?

If several people drop out beforehand and the decision is made to postpone the group, due to low numbers, the researcher will send you an email as soon as possible notifying you and the other participants that the group has been postponed due to low numbers. It is possible that people will not notify the researcher that they can no longer attend and will just not turn up on the day. If this happens, then a decision may be made to postpone the group at the start of the meeting. Whilst this would be unfortunate, it would be an unforeseen situation. Every effort will be made by the research team to avoid this.

How will the group be structured?

The researcher will ask you to sign a consent form to participate. As the group is being held online, a link to this will be emailed across to you at least one week before the meeting so that you can sign it electronically and return it to the researcher. When everybody has arrived in the online space, the researcher will facilitate introductions and lay out the ground rules for the group (at this point the researcher may mute everybody else’s microphones to reduce noise). You will then be provided with an opportunity to ask any questions about the BCTs and the example applications that are laid out in table 1.

Then, the researcher will start by asking the group the following question:

"Are there any other ways (or better ways) that the BCTs listed in table 1 could be applied at [redacted], that were not included in the information package?"

- First, you will be asked to think about this question privately. You are invited to think as **flexibly and creatively** as possible about how the BCTs could be put into practice on the wards at [redacted]
- You will then be invited to share your thoughts and other members of the group will be asked to do the same. This will be done in a 'round robin' format. This means that everybody will be invited to share one idea at a time, person by person, until everybody has said everything that they want to. As ideas are presented, a member of the research team will type them onto a virtual whiteboard in MS Teams so that everybody can see the new ideas.
- Then, you will be asked to openly discuss all the ideas that have been shared with group members. This is an opportunity to ask other group members about their suggestions and to clarify your understanding. At this stage, as a group, you may decide to combine some ideas if you agree that they are very similar.
- After the discussion, you will be asked to do **2 things**:
    - First, from the longer list of BCTs/applications (including the original ones from the information package and those added during the group discussion), you will be asked to vote on the **5*** BCT/applications that you believe would be the **most acceptable** to staff at [redacted] ranking them from 1 (most acceptable) to 5.
    - Then, you will be asked to repeat the same activity, but this time ranking the **5*** BCT/applications that you believe would be **easiest to put into practice** on the wards at [redacted] from 1 (most easy) to 5.
- After the group, the researcher will send you a summary of the information from these ranking exercises using email. You will be invited to comment on the information by replying to the email, but a response is not compulsory.

***** Any BCTs and applications that you do not rank will not be seen by the research team to be part of your response, so you <u>do not</u> need to attempt to rank these. Just focus on voting on the 5 that you consider to be the most important. This does not mean that other BCTs and example applications will not be considered by the research team when they are compiling the draft intervention. All BCTs will be considered; however, those that you and the other participants rank highly (top 5) will be prioritised.

What will happen to the data after the group?

After the group, the researcher will collate the information from the ranking exercises. Those BCTs and applications that were ranked highest by participants, will be reviewed first by the researcher and his supervisors when they are drafting the behaviour change intervention.

Further guidance about these activities will be provided by the researcher during the group. However, if you have any questions or concerns, please do not hesitate to contact the researcher: [redacted]

Thank you for reading this information package.
