# Supplementary material for: Selecting intervention content to target barriers and enablers of recognition and response to deteriorating patients: an online nominal group study
Source: BMC Health Serv Res. 2022 Jun 10;22:766. doi: 10.1186/s12913-022-08128-6 (PMC9186287; doi:10.1186/s12913-022-08128-6)
Supplement: Supplementary file 2 — Additional file 2. Facilitator’s guide for nominal groups. [file 12913_2022_8128_MOESM2_ESM.pdf]

Additional file 2 - facilitator's guide for nominal groups

DEveloping a Complex Intervention for DEteriorating Patients using Theoretical Modelling (DECIDE study)

Facilitator's guide for groups where nominal group technique will be applied

(Nursing staff and members of the [redacted] Deteriorating Patient Steering group)

Summary of facilitator roles

Facilitator 1 [redacted]	Lead the group including introductions, ground rules, and signalling transitions between the different activities.
Facilitator 2 [redacted]	Providing expert guidance on the appropriateness of the linkages between BCTs and applications proposed by the group during stage 2.
Facilitator 3 [redacted]	Providing expert guidance on the appropriateness of the linkages between BCTs and applications proposed by the group during stage 2.
Facilitator 4 [redacted]	Typing participant responses onto the virtual whiteboard/shared MS Word document in real time.<br>Taking paper notes and intermittent screenshots of the virtual whiteboard/shared MS Word document to ensure that key information is not lost if there is software/hardware failure.<br>Updating the ranking list of BCTs/applications, in real time, based on information from the virtual whiteboard/shared MS Word document.

Introduction and briefing (facilitator 1) – 15 minutes

- Open, welcome and thank participants for attending
- Ask all participants to say "hello" and to briefly introduce themselves (clarify the role of each supervisor, including the role of the health psychologists within the facilitation team)
- Clarify the nominal group process including the context, purpose, ground rules* and structure
- Offer the participants the opportunity to ask questions about the content of the information package e.g. to clarify the meaning of any BCTs (any questions may be deferred by DS to MC or JD to answer)
- Stress *"we are using this technique as it has been shown to allow everyone to contribute equally to feedback"*

**Ground rules:*

- Please respect one another's privacy by not discussing who attended or repeating anything that is said.
- If you do not understand a point made by another group member, be respectful in your inquiry
- Where the technology allows, keep video cameras switched on to facilitate a more personal feel to the group (opportunities for private work will be sign posted).
- Mute microphones when not speaking or participating in silent or private activities.
- Use the 'raise your hand' icon if you wish to speak within the group during the more open discussions in stage 2.
- Try not to get too pre-occupied with the language of the BCTs, the focus of discussion should be more on **how** the BCTs could be delivered.

- Whilst creativity and innovation are encouraged, request that participants try to keep their focus on ways of the delivering the BCTs rather than broader solutions (e.g. major organisational changes within the hospital or reforms to nursing education) to the example barriers given in the table.
- The barriers and enablers within the table are examples i.e. this is not an exhaustive list.
- Facilitators may interrupt to move the conversation on. This does not mean that the suggestion being made is not valuable, it will just be to ensure that we keep to time.

Stage 1 – individual responses (facilitators 1 and 4) (15 mins + 25 mins)

- Facilitator 1 poses the opening question:

"Are there any other ways (or better ways) that the BCTs listed in table 1 could be applied at [redacted] *that were not included in the information package?"*

- Participants are asked to privately and silently consider alternate ways in which the BCTs in table 1 (3rd column) could be applied (put into action) at [redacted] (other than the examples in column 5 – *though suggesting an amendment to, or an elaboration of, one of these examples is quite acceptable*). They do not need to come up with ideas for every BCT, just those that speak to them the most. *Every group member will have the chance to share one idea minimum. We may have the opportunity to cycle around the group more than once and hear several ideas; however, we may not (it really depends on time). As such, if participants have several suggestions, ask that they share them in the order of priority so that we will have heard the most important points from their perspective.*
- Participants may want to jot ideas down on a piece of paper or on a separate notes page on their computer so that they do not forget.
- Participants are encouraged to think as broadly, creatively, and as 'out of the box' at this stage as they can (scaling down will come in later activities if appropriate).
- Participants are told that they have 15 minutes, but that they will be prompted when they are due back into the virtual space.

- After 15 minutes, facilitator 1 brings the group out of their silent phase and asks each person in turn to give just 1 idea that they came up with in response to the question. *Note: when the participants are sharing their ideas, ask that they clarify which BCT their idea relates to so that this can be captured on the whiteboard/shared MS Word document. They do not need to read out the barrier – just to clarify the number and the BCT to which their idea relates.*
- When offering suggestions during the 'round robin' exercise remind participants to speak descriptively without lots of evaluation (e.g. rationale) or opinion.

- Participants are asked not to repeat an idea that has already been given in the information package or by another participant during the group, but they may present an idea if it represents a “variation on the same theme” i.e., it extends an existing idea or involves a different level of emphasis.
- Whilst the participants are giving their response, facilitator 4 enters the ideas onto a virtual whiteboard/shared MS Word document (that all group members can see). Each idea is numbered (13,14,15…) for ease of reference later **[Note: numbering should start at 13 as 1-12 are the number labels given to the existing examples within the information package].** *Points should be typed into text boxes so that they can be manipulated. A landscape A4 sheet should be used in MS Word to make it easier for participants to read the information* (see appendix 1 for further guidance).
- This round robin exercise continues until all ideas have been offered up and recorded or time runs out for this activity.
- Once an idea has been added to the virtual whiteboard/shared MS Word document it belongs to the whole group and decision making about grouping ideas etc. should reflect this.

Stage 2 – clarification and consolidation of responses (facilitators 1, 2, 3 & 4) (25 minutes)

- Participants are invited by facilitator 1 to seek clarification from other participants about their suggestions/ideas.
- Participants are then invited to edit their whiteboard/shared MS Word document by merging suggestions/ideas that overlap. Number labels applied in stage1 may be used for ease here. If 2 points are merged, a new number may be allocated to the resultant point*. Note: the facilitator must be careful at this stage not to comment on or evaluate the points/decisions made by the participants. However, DS can consider asking open questions to seek further clarification if the link between the BCT and application seems tenuous e.g. can you explain how that application relates to the BCT?*

*****15 to 20-minute comfort break for participants here – during the comfort break all facilitators enter another MS Teams space and focus on the following:***

- Adding BCT/applications to the ranking sheet so that it can be shared (see appendix 2 for further guidance) – facilitator 4.
- Prioritising where the BCT and suggested application do not clearly link and need to be adjusted - facilitators 2 & 3.
- Agreeing how to feedback adjustments to the participants.

- Facilitators 2 and 3 invited to comment on the linkages between the BCTs and the applications generated during stage 1 (this might include gently highlighting where a BCT and application do not appear to align or where there appears to be confusion about the meaning of a BCT).

Stage 3 – ranking exercises (20 minutes - 10mins + 10mins

- The link for the ranking document in Qualtrics is posted into the Teams discussion thread (having been updated in real time by facilitator 4) - [redacted]
- Participants are asked to access the poll and do two things:
- From the longer list they should **rank 5 of the BCTs and applications** that they feel would be the **most acceptable** to ward staff.
- When they click on the link it will take them to a list of the BCTs and applications from the table plus any additional ones that were suggested by group members (these will have been added on during the discussion and will appear at the bottom of the list). They should rank from 1 (most acceptable) to 5. This can be done by typing the number (1-5) in the little box above the relevant point (see pictures in appendix 1). *
- Highlight to the participants that the numbers they see next to the points below reflect either the number from the table within the information package, or the number allocated on the virtual whiteboard/shared MS Word document in Microsoft Teams. As such, they should **not** let this number influence how they rank the items, this is just a label to help them identify each of the items from the information that they already have.
- They should then repeat this activity according to how easily they believe the BCT/applications could be put into practice at [redacted] from 1 (easiest) to 5.

*Remind participants that any BCTs and applications that they do not rank will not be seen by the research team to be part of their response, so they <u>do not</u> need to attempt to vote on these (Qualtrics will block them from doing so). They should just focus on ranking the 5 that they consider to be the most important. This does not mean that other BCTs and example applications will not be considered by the research team when they are compiling the draft intervention. All BCTs will be considered; however, those that they rank highly (top 5) will be prioritised.

Closing remarks

Inform participants that a summary of the ranking data will be circulated via email. Invite participants to comment on the accuracy by responding to the email if they want to.
Thank participants again for their contributions.

List of abbreviations:

BCTs	Behaviour Change Technique/s
MS	Microsoft®
