# Supplementary material for: Selecting intervention content to target barriers and enablers of recognition and response to deteriorating patients: an online nominal group study
Source: BMC Health Serv Res. 2022 Jun 10;22:766. doi: 10.1186/s12913-022-08128-6 (PMC9186287; doi:10.1186/s12913-022-08128-6)
Supplement: Supplementary file 3 — Additional file 3. Professional role of participants attending nominal groups. [file 12913_2022_8128_MOESM3_ESM.pdf]

Additional file 3 – professional role of participants attending nominal groups

NGT 1 - leadership group participants (number of participants in this role who attended)	NGT 2 - clinical group participants (number of participants in this role who attended)
– Deputy chief nurse (1)<br>– Consultant in intensive care medicine (1)<br>– Quality improvement practitioner (1)<br>– Clinical-academic physiotherapist (1)<br>– Senior nurse - education (2)<br>– Senior nurse - critical care outreach (4)<br>– Lead clinical nurse specialist (1)<br>– Ward matron (1)	– Charge nurse/ward manager (2)<br>– Clinical nurse specialist (1)<br>– Deputy charge nurse (1)<br>– Staff nurse (2)<br>– Healthcare assistant (1)
