# Supplementary material for: Selecting intervention content to target barriers and enablers of recognition and response to deteriorating patients: an online nominal group study
Source: BMC Health Serv Res. 2022 Jun 10;22:766. doi: 10.1186/s12913-022-08128-6 (PMC9186287; doi:10.1186/s12913-022-08128-6)
Supplement: Supplementary file 4 — Additional file 4. The number and labels of the Behaviour Change Techniques (BCTs) mapped from each of the 9 Theoretical Domains Framework (TDF) domains of high importance. [file 12913_2022_8128_MOESM4_ESM.pdf]

Additional file 4 – the number and labels of the Behaviour Change Techniques (BCTs) mapped from each of the 9 Theoretical Domains Framework (TDF) domains of high importance

**TDF domains of high importance**	**Number of BCTs mapped from the domain**	**BCT labels (from the mapping tool in appendix 10)** *BCTs in red were mapped from two different TDF domains.*
Knowledge	4	– Antecedents<br>– Biofeedback<br>– Feedback on behaviour<br>– Health consequences
Social Professional Role and Identity	1	– Social, support or encouragement (general)
Beliefs about Consequences	10	– Anticipated regret<br>– Comparative imagining of future outcomes<br>– Covert conditioning<br>– Covert sensitisation<br>– Emotional consequences<br>– Pros/cons<br>– Salience of consequences<br>– Social and environmental consequences<br>– Threat<br>– Vicarious reinforcement
Reinforcement	17	– Anticipation of future rewards or removal of punishment<br>– Classical conditioning<br>– Counter conditioning<br>– Differential reinforcement<br>– Discrimination training<br>– Extinction<br>– Incentive<br>– Material reward<br>– Negative reinforcement<br>– Non-specific reward<br>– Punishment<br>– Response cost<br>– Self-reward<br>– Shaping<br>– Social reward<br>– Thinning<br>– Threat
Intentions	2	– Behavioural contract<br>– Commitment
Goals	5	– Action planning<br>– Goal setting (behaviour)

		– Goal setting (outcome)<br>– Review behavioural goals<br>– Review of outcome (goals)
Memory, Attention and Decision Processes	3	– Action planning<br>– Prompts/cues<br>– Self-monitoring of behaviour
Environmental Context & Resources	5	– Avoidance/changing exposure to cues for the behaviour<br>– Discriminative cue<br>– Prompts/cues<br>– Re-structuring the physical environment<br>– Re-structuring the social environment
Social Influences	10	– Identification of self as a role model<br>– Information about others' approval<br>– Modelling or demonstrating the behaviour<br>– Re-structuring the social environment<br>– Social comparison<br>– Social reward<br>– Social support (emotional)<br>– Social support (practical)<br>– Social support or encouragement (general)<br>– Vicarious reinforcement
